# Tuning Bond Exchange Rate to Control Long-Time Mobility and Creep in Vitrimer-like Networks

**DOI:** 10.1021/acsapm.6c00856

**Published:** 2026-08-12

**Authors:** Ablajan Mamat, Rakan Alrashdan, Tyler Eakin, Harsh Pandya, Patrick Cuddihy, Kevin Barz, Michel Cloitre, Fardin Khabaz

**Affiliations:** † School of Polymer Science and Polymer Engineering, 1076 The University of Akron, Akron, Ohio 44325, United States; ‡ Department of Chemical, Biomolecular, and Corrosion Engineering, 354236 The University of Akron, Akron, Ohio 44325, United States; § Molecular, Macromolecular Chemistry, and Materials, ESPCI Paris, CNRS, PSL Research University, Paris 75005, France

**Keywords:** vitrimers, time−temperature superposition, dynamics, rheology, creep

## Abstract

Vitrimers relax stress through a combination of polymer mobility and associative bond exchange, but connecting exchange kinetics to microscopic dynamics and macroscopic rheology remains challenging. Many simulation schemes do not allow independent control of the exchange rate. Here we utilize a molecular dynamics–Monte Carlo simulation technique for associative networks in which the exchange attempt interval is an explicit, independently tunable control parameter, enabling systematic scans of exchange kinetics at fixed network architecture and interactions. Using mean-squared displacement and creep compliance as indicators of the microscopic dynamics and macroscopic rheology, we show that slowing the bond exchange rate delays the onset of long-time mobility and terminal creep without significantly altering short-time local dynamics, indicating a separation between segmental mobility and topology-change relaxation. Time–temperature superposition is obeyed only partially: no single set of shift factors collapses the full microscopic and rheological responses of the associative networks. Direct analysis of the bond-memory relaxation time reveals an approximately Arrhenius temperature dependence, whereas the permanent-network shift factors follow WLF behavior. The resulting mismatch between the polymer-controlled and exchange-controlled clocks causes their relative positions to change with temperature, thereby controlling the crossover from elastic or plateau response to long-time mobility and terminal creep.

## Graphical abstract

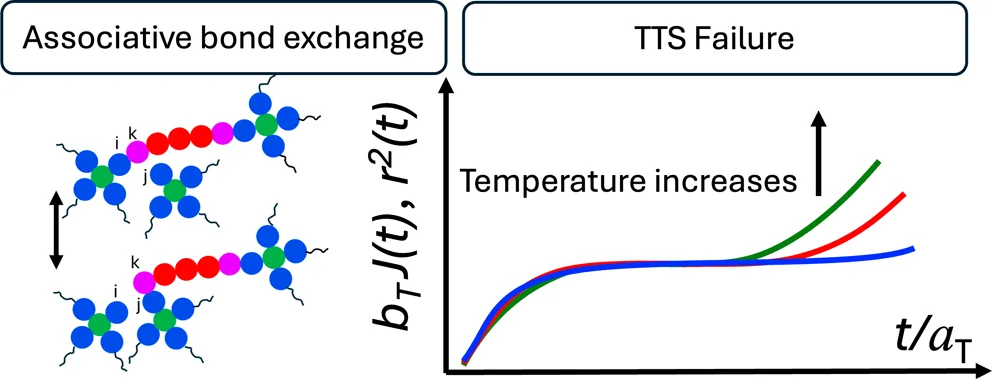

## 1 Introduction

Dynamic covalent polymer networks, often termed covalent adaptable networks (CANs), were introduced to reconcile the durability of thermosets with the reprocessability of thermoplastics by embedding exchangeable covalent chemistry into an otherwise cross-linked architecture.^1–5^ Among CANs, vitrimers are the prototypical associative class: cross-links exchange via bond-swap reactions that preserve cross-link density while permitting topology fluctuations, stress relaxation, and at sufficiently long times, macroscopic flow.^2–4^ This mechanism has enabled recyclable high-performance polymers, reprocessable composites, and self-healing materials across diverse chemistries and processing routes.^6,7^

A recurring theme in vitrimer design is that exchange chemistry, network architecture, and microstructure are tightly coupled.^2,3,8,9^ Associative exchange can be catalyzed or catalyst-free and can coexist with permanent cross-links or physical associations, producing hybrid networks with tailored creep resistance, toughness, and malleability.^6,10,11^ Moreover, vitrimer chemistries that introduce polarity or specific interactions can promote self-assembly or phase separation of exchangeable motifs, yielding heterogeneous morphologies that strongly shape long-time relaxation.^12–19^ Recent works have also clarified how network architecture and catalyst-free exchange control relaxation and high-rate dissipation in epoxy- and silicone-based vitrimers.^20–22^

From a rheological standpoint, vitrimers often sit at the intersection of at least two coupled “clocks”: (i) polymeric relaxation (segmental friction, strand/Rouse relaxation, and entanglement dynamics when present) and (ii) bond exchange kinetics that enable constraint release and topology rearrangement.^13,23–26^ Because these mechanisms can carry distinct temperature dependences—often WLF-like scaling for polymeric friction near glassy regimes versus Arrhenius-like activated exchange—the relaxation spectrum can change *shape* with temperature rather than simply shift.^25,27–31^ Consequently, vitrimers are frequently thermo-rheologically complex: different observables (or different time/frequency windows) may be dominated by different mechanisms.^16,25,26^ These features directly challenge the routine application of time–temperature superposition (TTS).^27,28,32^ In its standard form, TTS assumes thermo-rheological simplicity, i.e., that changing *T* primarily rescales time by a single horizontal shift factor *a_T_* (and sometimes a vertical factor) without altering the spectrum shape, so that for a linear-response observable *X* one expects *X*(*t*; *T*) ≈ *X*(*t*/*a_T_*; *T*_ref_) over the full accessible window.^27,28^ When this assumption fails, one observes window-dependent shifting, protocol/observable-dependent shifts, and representation-independent diagnostics (e.g., van Gurp–Palmen plot) that do not collapse.^26,28,32^

Several strategies have been proposed to deal with TTS failure in vitrimers as a consequence of the existence of relaxation processes of different physical nature within the same experimental window.^25,26,29–31,33,34^ A pragmatic approach is to apply different shift factors to different parts of the spectrum, associating short-time/high-frequency response with segmental mobility and long-time/low-frequency response with exchange-controlled relaxation.^33,35^ A more analytical route is to decompose the response into two families of relaxation modes with distinct temperature dependencies and recombine them to reconstruct spectra at arbitrary temperature.^29^ Complementarily, tube-based time-marching frameworks have been modified to incorporate exchange dynamics explicitly and rationalize how segmental and exchange clocks jointly determine linear viscoelasticity.^36^ Together with sticky/associative network pictures in which chain relaxation and exchange-mediated constraint release compete,^13,23–25^ these works reinforce the central message that a single universal master curve is not generally expected once multiple relaxation times overlap within the measured window.^29,30,33^

Complementing experiments, theoretical and computational frameworks have been developed to interpret vitrimer relaxation as a competition between chain modes and exchange-mediated constraint release.^13,23–25,37–39^ Continuum and constitutive descriptions capture macroscopic stress relaxation and flow under bond exchange,^40–42^ while coarse-grained and molecular models reveal how local bond topology, defects, and heterogeneous environments provide pathways for stress relaxation and healing.^14–18,43^ Molecular simulation is particularly valuable because exchange attempt rates and activation barriers can be tuned independently of nonbonded interactions, allowing direct tests of whether exchange alters equilibrium structure/thermodynamics or primarily reprograms long-time dynamics.^44–47^ Hybrid Molecular Dynamics–Monte Carlo (MD–MC) simulations and related approaches have been used to quantify thermodynamics, packing, and dynamics in model vitrimers and CANs,^19,48–55^ to interrogate microstructure–dynamics links,^16–18^ and to access nonequilibrium signatures such as intermittent stretch fluctuations and flow-driven rearrangements that can dominate long-time response.^56^ Recent coarse-grained nonequilibrium simulations have introduced reactive bead–spring models for associative networks and linked bond exchange to extensional rate-thinning and stretch intermittency.^56,58^ Furthermore, simulation work has highlighted how reversible cross-links can impact cohesion, density, and microphase separation in associative vitrimers.^59–61^

A key unresolved issue is when the vitrimer response is governed by the coexistence of polymer-controlled and exchange-controlled processes that yields temperature-dependent reshaping of the spectrum.^13,25,29,30,33^ In experiments where exchange effects are indirectly inferred from macroscopic spectra, similar viscoelastic signatures can arise from distinct microscopic mechanisms.^25,26^ For example, an apparent “single” terminal relaxation time may reflect exchange-controlled topology rearrangement in which bond swapping governs the approach to terminal flow, or instead polymer-controlled relaxation in which segmental mobility sets the time scale while exchange is effectively inactive within the probed window.^25,26^ Likewise, a second slow mode can originate from a distribution of local exchange rates and environments (e.g., catalyst heterogeneity, variable local polarity, or microphase-separated sticker-rich domains), or from network-architectural heterogeneity (dangling ends, loops, ineffective cross-links) that broadens the spectrum even at fixed chemistry.^25,26^ Discriminating among these possibilities generally requires combining multiple protocols and linking macroscopic response to microscopic measures of bond persistence and chain mobility,^25,26^ motivating controlled simulations that vary *T* and exchange kinetics independently while tracking both equilibrium metrics and particle-scale dynamics.^19,37,48–50^ However, in MD simulations, tuning the bond exchange rate while keeping the number of bonds constant constitutes a real challenge. It has been recently achieved using a three-body potential energy for describing interactions in model vitrimers,^14,37^ in which the rate of bond exchanges is embedded in the potential form using a theoretical parameter. In combination with a microscopic statistical mechanical theory^39^ it was shown that the bond-exchange kinetics influence segmental relaxation and affect the glass-transition temperature.^62,63^

In our prior work,^48,49^ we showed that in the limiting regime of extremely fast bond exchange, a separation of time scales can render vitrimer dynamics approximately thermorheologically simple and compatible with TTS. The present work moves beyond that fast-exchange limit by targeting the intermediate regime where polymer relaxation and exchange-mediated topology rearrangement occur on comparable time scales, leading to competing clocks and possible breakdown of TTS in highly cross-linked networks such as epoxy resins. We also demonstrated that modest applied stress can activate exchange events even in cross-linked networks.^64,65^ This two-clock dynamics is present by design in the model. Here, our objective is to determine how their relative placement within the accessible simulation window controls structure, mobility, creep, and the success or failure of time–temperature superposition. In this way, the simulations will provide a controlled regime map for when exchange kinetics becomes dynamically relevant relative to molecular motion. Here we target the experimentally relevant intermediate regime where exchange and polymer relaxation compete within the same observation window, and we address a key limitation of many simulation studies in which exchange kinetics are tied to thermodynamic parameters or the MC move design. Using a bead–spring network with associative exchange implemented via a hybrid MD–MC scheme,^24,48^ we treat the swap-attempt interval as an explicit, independently tunable parameter and vary it alongside temperature at fixed interactions and network architecture. We additionally determine the bond-memory relaxation time directly from the bond correlation function, allowing the temperature dependence of the exchange-controlled clock to be distinguished from the WLF-like polymer-relaxation clock of the permanent network. We show that once exchange contributes appreciably, no single *a*_*T*_ collapses the full time response, even though equilibrium structure and thermodynamics remain essentially unchanged.

## 2 Simulation Details and Model

We consider a three-dimensional polymer network composed of short oligomers and tetra-functional cross-linkers. The simulation box contains *N*_p_ = 2880 linear oligomers with five beads per chain and *N*_c_ = 1440 tetra-functional cross-linkers. Each bead has mass *m* and an effective diameter σ, which defines the unit length through the Lennard-Jones (LJ) interaction. Nonbonded pairs of beads interact via a truncated and shifted LJ potential

1 $$U_{\mathrm{LJ}}\left(r\right)=4\varepsilon \left[{\left(\frac{\sigma }{r}\right)}^{12}-{\left(\frac{\sigma }{r}\right)}^{6}-{\left(\frac{\sigma }{r_{c}}\right)}^{12}+{\left(\frac{\sigma }{r_{c}}\right)}^{6}\right]H\left(r_{c}-r\right)$$

where *H*(*r*) is the Heaviside step function, defined as *H*(*r*) = 1 for *r* > 0 and *H*(*r*) = 0 for *r* ≤ 0, with cutoff distance of *r*_c_ = 2.5σ,^48^ and ε sets the characteristic energy scale. Beads that are chemically bonded interact via the finitely extensible nonlinear elastic (FENE) potential^45^

2 $$U_{\mathrm{FENE}}\left(r\right)=-\frac{1}{2}kR_{0}^{2}\ln\left[1-{\left(\frac{r}{R_{0}}\right)}^{2}\right]$$

with spring constant *k* = 30 ε/σ^2^ and maximum bond extension *R*_0_ = 1.5σ. Throughout this work, we use reduced units defined by the diameter of the bead σ for length, the energy well-depth of the LJ potential ε for energy, and the mass of the bead *m*. Considering these scales, the natural time scale is $\tau =\sqrt{m\sigma^{2}/\varepsilon }$ . Throughout this paper, all the parameters are presented in dimensionless units.

The initial configuration is prepared by randomly mixing the polymer chains and cross-linkers in a cubic box with periodic boundary conditions and equilibrating the melt at *P* = 0 and *T* = 1. A chemically connected network is then constructed in two steps. First, we generate a cross-linked state in which each cross-linker forms four bonds to chain ends. For every bond that is formed, the cross-linker reacting bead selects the nearest available polymer end, considering that two reacting beads from one cross-linker cannot connect to the ends of a chain. This procedure continues until all cross-linkers have four bonds and both ends of every chain are linked. Second, we remove 5% of newly created bonds. Cross-linkers are selected for removal under the constraint that no polymer chain loses cross-linkers at both ends, and at least one chain end must remain connected to a cross-linker. The latter is carried out because free cross-linker beads can initiate associative bond exchanges in the model. Immediately after bond construction and removal, some bonds are overstretched. To remove this artificial strain, the FENE bonds are temporarily replaced by harmonic springs, and the network is relaxed at constant temperature until the average bond length converges to *r* = 0.96. Afterward, all harmonic bonds are switched back to the FENE form, resulting in a mechanically relaxed isobaric–isothermal ensemble at a temperature of *T* = 1.0 and pressure of *P* = 0 with an equilibrium bond length of 0.96.

The bond exchange events between cross-linkers and the end points of the strands are introduced through a hybrid MD–MC scheme. Only free cross-linking sites, hereafter referred to as defects, are allowed to initiate exchange moves, as illustrated in Figure 1A. We choose a small defect fraction (5%) to ensure a controlled population of free junctions that can initiate associative swaps while keeping the network close to fully connected; this avoids a trivial no-exchange limit caused by the absence of available partners. At prescribed time intervals *t*_Exch_, a trial associative swap is generated in which a free cross-linking site *j* replaces a bonded cross-linking site *i* at chain end *k*. Because the number of locally eligible exchange partners can differ before and after a trial swap, the exchange step is implemented using a Metropolis–Hastings acceptance rule. For a proposed move, we count the number of eligible forward candidates, *N*_f_, in the current topology and the number of eligible reverse candidates, *N*_r_, in the trial topology generated by the same geometric cutoff and bonding constraints. For uniform selection among locally eligible partners, the proposal ratio is *q*_reverse_/*q*_forward_ = *N*_f_/*N*_r_, where *q*_forward_ and *q*_reverse_ are the probabilities of proposing the forward and reverse moves, respectively. The acceptance probability is therefore $P_{\mathrm{acc}}=\min\left[1,\exp\left(-\frac{\Delta U_{\mathrm{Exch}}}{T}\right)\frac{N_{f}}{N_{r}}\right]$ . This ratio of accounts for cases in which the forward and reverse configurations have different numbers of possible exchange partners and ensures detailed balance for the MC exchange step. The fixed interval *t*_Exch_ controls how often this detailed-balance-preserving exchange kernel is applied.

**Figure 1:**
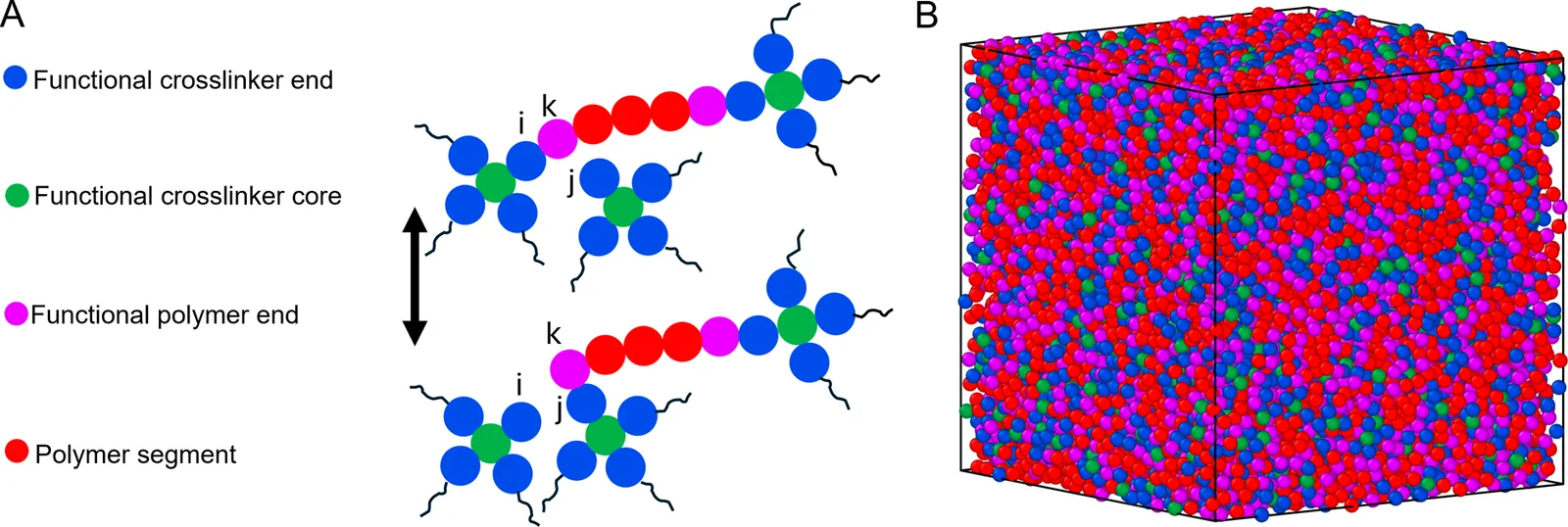
(A) Schematic of the bond exchange rule used in the MC bond exchange step, *A* – *B*_*i*_ + *B*_*j*_^free^ ⇌ *A* – *B*_*j*_ + *B*_*i*_^free^. A free cross-linker bead *j* can exchange a dynamic bond with a cross-linker bead, *i*, that is already bound to a polymer end, *k*: when *j* encounters the (*i, k*) pair, the bonding partner at the polymer end is swapped so that the polymer end becomes bound to *j*, while bead *i* becomes free. (B) Representative snapshot of the simulated polymer network configuration.

In the present framework, *t*_Exch_ is the imposed interval between exchange attempts and should be distinguished from the realized bond-exchange or bond-memory relaxation time, which also depends on temperature, local geometry, and segmental mobility through the acceptance probability. A direct comparison with an experimental system can be made in principle by matching dimensionless ratios of time scales, for example $\frac{\tau_{\mathrm{bond}}^{\mathrm{sim}}}{\tau_{\mathrm{seg}}^{\mathrm{sim}}}∼\frac{\tau_{\mathrm{ex}}^{\exp}}{\tau_{\alpha }^{\exp}}$ , where τ_bond_^sim^ may be obtained from the decay of the bond correlation function, τ_seg_^sim^ is a segmental or cage-relaxation time measured in the same simulation, and τ_ex_^exp^/τ_α_^exp^ is the corresponding experimental ratio from long-time exchange-controlled relaxation and segmental/α relaxation. Thus, the values of *t*_Exch_ should be interpreted as a controlled scan from frequent to infrequent exchange attempts relative to molecular motion, rather than as direct assignments of a chemistry-specific activation barrier. The apparent activation energy of the realized bond–memory process is instead obtained independently from the temperature dependence of τ_bond_.

Following network construction, a final relaxation protocol is applied: systems are first equilibrated in the *NPT* ensemble at *T* = 1 to remove residual stresses and adjust the density, and subsequently relaxed in the *NVT* ensemble at the same temperature to stabilize the bonded network prior to production runs. For each *t*_Exch_, five independent replicas are used to determine the properties. The time evolution of the networks is generated using a time step Δ*t* = 0.005 and applying periodic boundary conditions.

## 3 Results and Discussion

### 3.1 Thermodynamics of Networks with Different Bond Exchange Rates

The quenching simulation was performed starting from *T* = 1.0 using 16 successive cooling stages; in each stage, the temperature was decreased by 0.05, and the networks were evolved for 10^7^ steps, corresponding to a quenching rate of *q̇* = 10^–6^ in reduced units. Figure 2A shows the volume of the system scaled by the number of beads, *V*, as a function of reduced temperature, *T*, for networks with different exchange-attempt intervals, *t*_Exch_. A change in slope is observed at about *T* = 0.43, corresponding to the glass transition. This value is slightly higher than the typical glass transition temperature of linear bead–spring melts due to dense network architecture.^66^ *T*_g_ is also determined from the temperature dependence of the thermal expansion coefficient, α (inset of Figure 2A), which is defined as $\alpha =\frac{1}{V}{\left(\frac{\partial V}{\partial T}\right)}_{P}$ , and the resulting *T*_g_ is then mapped back onto the *V*–*T* curve. Note that here, *T*_g_ denotes the volumetric glass transition, and should be distinguished from the topology-freezing or terminal-relaxation temperature, which is expected to be more directly controlled by bond-exchange kinetics.^4,62,63^

**Figure 2:**
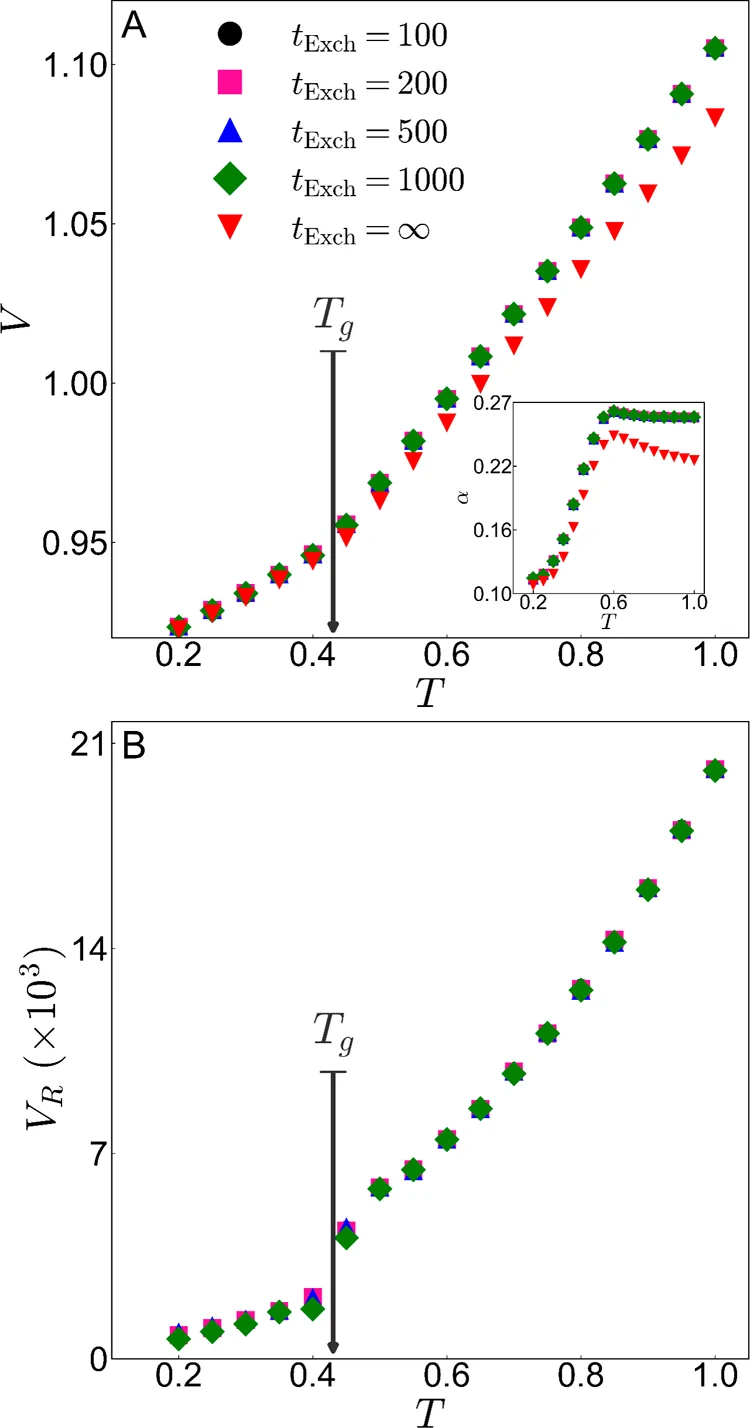
Temperature dependence of (A) the scaled volume, *V*, and (B) the relative volume change *V*_R_, $V_{R}=\frac{V-V_{p}}{V_{p}}$ , of polymer networks with different exchange-attempt intervals, *t*_Exch_, as a function of *T. V*_p_ is the reference volume obtained through the permanent network. The arrow indicates the glass transition temperature, *T*_g_, identified from the change in slope of *V* vs *T*. The inset in A shows the corresponding thermal expansion coefficient, α, as a function of *T*.

Below *T*_g_, the *V*–*T* curves are nearly identical for all *t*_Exch_, indicating that associative exchange does not measurably alter the glassy packing state over the simulation time scale. Above *T*_g_, the networks with finite *t*_Exch_ exhibit a small increase in specific volume relative to the permanently cross-linked network (*t*_Exch_ = ∞). However, the curves for *t*_Exch_ = 100, 200, 500, and 1000 remain nearly superimposed. Thus, enabling associative exchange produces a modest volumetric effect in the rubbery regime, but this effect does not vary systematically with the exchange-attempt interval over the range investigated. The normalized relative volume, *V*_R_ = (*V* – *V*_p_)/*V*_p_, where *V*_p_ is the specific volume of the permanent network, is used to isolate this small exchange-associated contribution from the baseline thermal expansion (Figure 2B). In the glassy regime, *V*_R_ ≈ 0 for all *t*_Exch_. Above *T*_g_, *V*_R_ becomes positive and increases with temperature, indicating a modest excess volume in the associative networks relative to the permanent network. Nevertheless, the *V*_R_ curves for the different finite exchange-attempt intervals nearly overlap. Therefore, the excess volume is primarily associated with the presence of topology exchange rather than with the frequency at which exchange moves are attempted.

To examine the effect of the kinetics of the bond exchange reaction on the structure of networks, the pair distribution function, *g*(*r*), between the central cross-linker beads at two representative temperatures of *T* = 0.8 and *T* = 0.3, which correspond to rubbery and glassy regimes, respectively, over a wide range of *t*_Exch_ has been determined (Figure 3). At both temperatures, *g*(*r*) exhibits weak correlation in the spatial distribution of the cross-linkers, and this suggests that the distribution of the cross-linkers in the system is mostly random, except for a few minor peaks at *r* ≈ 2–3, followed by weakly damped oscillations. At distances *r* > 5, *g*(*r*) approaches unity, converging to a fully random distribution regardless of the *t*_Exch_. In addition, the weak ordering of the cross-linkers at small length scales is more evident at low temperatures than at high temperatures.

**Figure 3:**
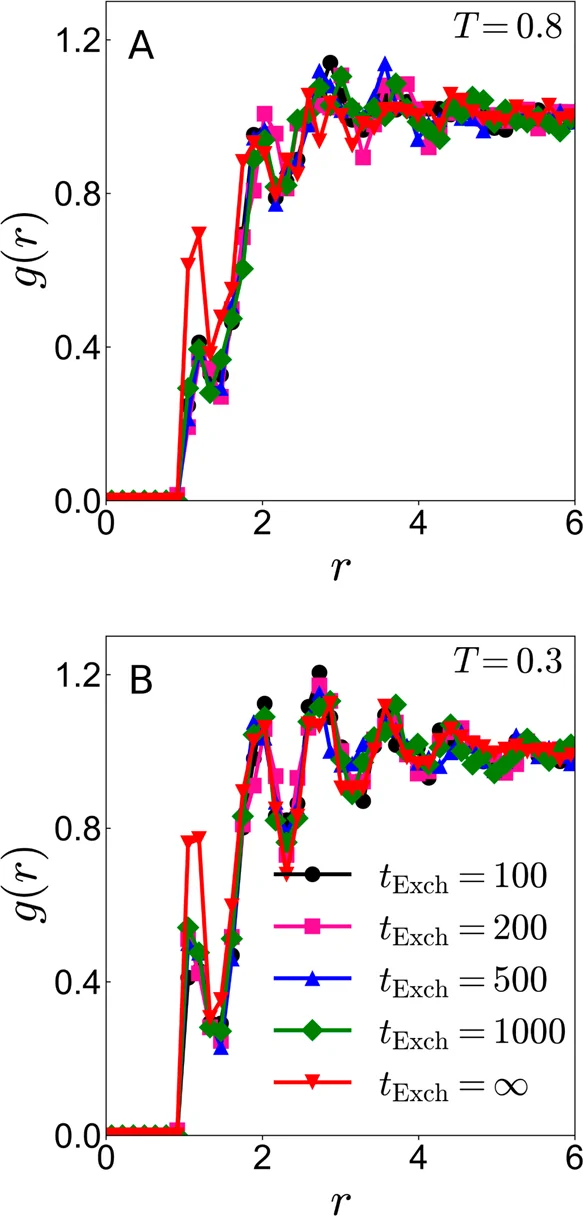
Radial distribution function, *g*(*r*), for networks at two reduced temperatures: (A) *T* = 0.8 and (B) *T* = 0.3. Curves correspond to different exchange-attempt intervals, *t*_Exch_.

Notably, at fixed *T*, the radial distribution functions corresponding to different *t*_Exch_ values nearly overlap across the entire range of *r*. The weak dependence on *t*_Exch_ indicates that enabling associative swaps primarily alters pathways and time scales of relaxation rather than equilibrium packing; at fixed interactions and density, local structure is governed mainly by *T* and connectivity, while exchange kinetics controls how rapidly the network explores topologies. This weak dependence of *g*(*r*) on exchange kinetics is consistent with prior theoretical treatments in which bond exchange modifies relaxation dynamics while leaving the equilibrium pair structure only weakly perturbed.^39^

We next examine the kinetics of bond exchange by determining the number of accepted MC moves at each temperature and exchange-attempt interval, *t*_Exch_, as shown in Figure 4A,B. For all values of *t*_Exch_, the total number of accepted exchanges, *N*_total_, increases with temperature, spanning more than 2 orders of magnitude from the glassy to the rubbery regime. At every temperature, the curves are ordered as *t*_Exch_ = 100 > 200 > 500 > 1000, showing that the cumulative number of topology rearrangements is controlled by how frequently the MC exchange procedure is applied.

**Figure 4:**
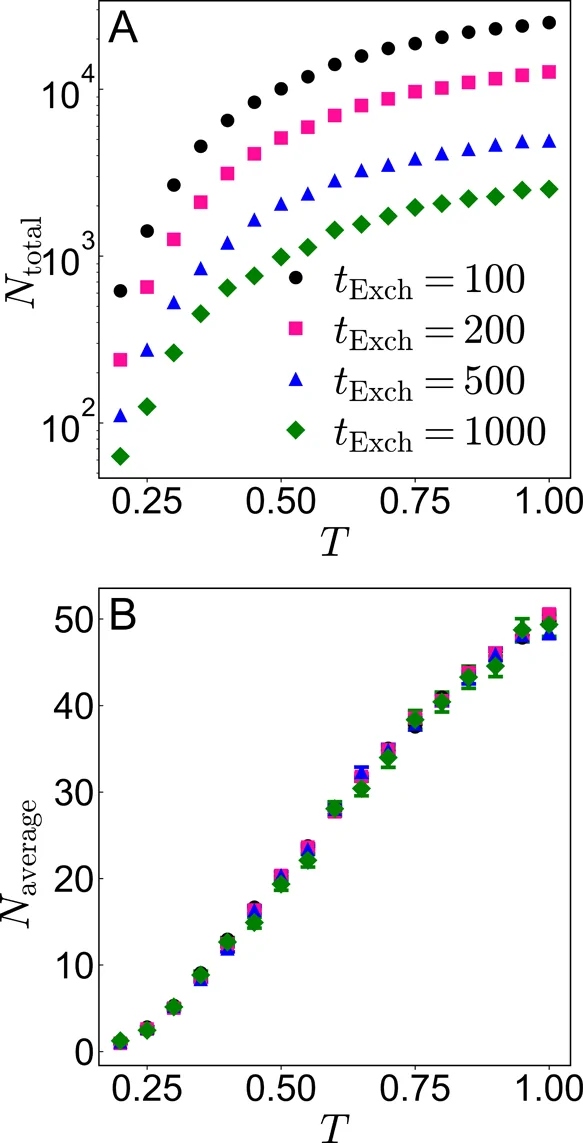
Temperature dependence of (A) the total number of accepted exchanges, *N*_total_, and (B) the average number of accepted exchanges during each MC exchange step, *N*_average_, as functions of reduced temperature *T* for different exchange-attempt intervals, *t*_Exch_.

Figure 4B shows the average number of accepted exchanges during each application of the MC exchange procedure, *N*_average_. For all values of *t*_Exch_, *N*_average_ increases approximately linearly with *T*, indicating that increasing temperature increases the number of geometrically and energetically favorable exchange moves. The data for the different exchange-attempt intervals nearly overlap, demonstrating that the number of accepted moves during a given exchange step is governed primarily by temperature and local molecular mobility and is essentially independent of *t*_Exch_ over the range investigated.

The strong separation among the *N*_total_ curves therefore arises mainly from the number of times the exchange procedure is applied during a fixed simulation interval. Smaller *t*_Exch_ produces more exchange steps and, consequently, a larger cumulative number of accepted exchanges, even though the average number accepted during each exchange step remains nearly unchanged. Below *T*_g_, accepted exchanges are reduced but remain nonzero. However, restricted segmental mobility limits the extent to which these local swaps generate large-scale topology relaxation and long-time transport.

### 3.2 Dynamics

We next examine how the bond-exchange time scale affects network mobility at both microscopic and macroscopic levels. Figure 5A–D show the mean-squared displacement (MSD), ⟨Δ*r* ^2^(Δ*t*)⟩, computed from the trajectories of the central beads of the cross-linkers at different temperatures in the canonical ensemble. At low temperature (Figure 5A), all networks exhibit similarly limited motion over the accessible time window. As the temperature increases above *T*_g_ (Figure 5B–D), the permanently cross-linked network (*t*_Exch_ = ∞) approaches a rubbery plateau because its topology remains fixed. In contrast, the associative networks exhibit a plateau or shoulder followed by an intermediate subdiffusive regime, approximately characterized by ⟨Δ*r* ^2^⟩ ∼ Δ*t* ^0.5^. At longer times, the faster-exchanging networks cross over toward quasi-diffusive motion, ⟨Δ*r* ^2^⟩ ∼ Δ*t*, consistent with long-time transport enabled by bond-exchange-mediated topology rearrangements. Increasing *t*_Exch_ prolongs the plateau regime and delays this long-time crossover. The quasi-diffusive regime becomes increasingly apparent at higher temperatures, particularly for *t*_Exch_ = 100 and 200, whereas for slower exchange it may remain beyond the accessible simulation window. Thus, bond exchange activates topology fluctuations that accelerate the crossover from caging to long-time transport. When *t*_Exch_ becomes much longer than the strand-relaxation time, exchange-mediated terminal relaxation is not observed within the accessible simulation window, and the response approaches the permanent-network limit.

**Figure 5:**
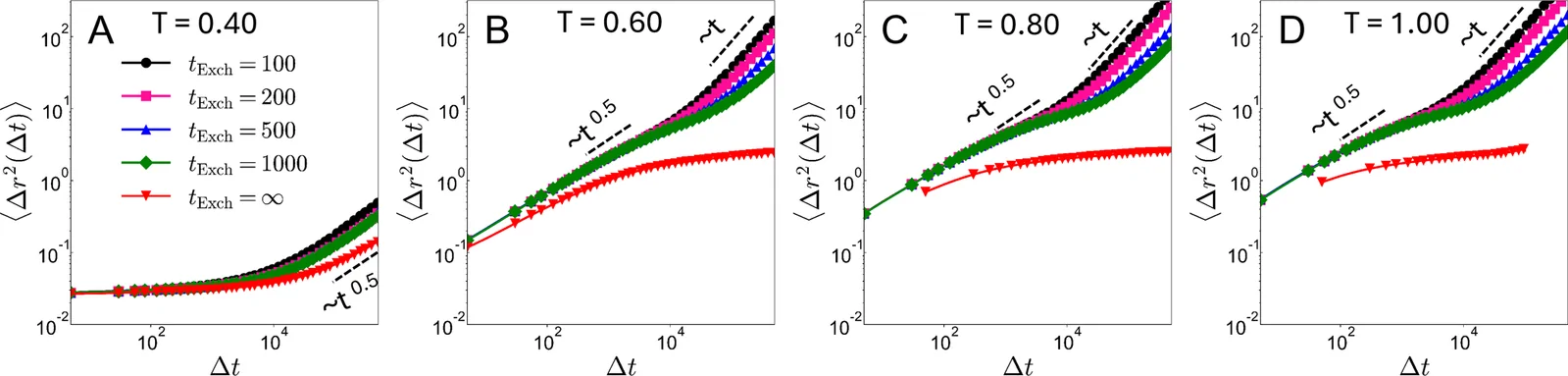
Mean squared displacement (MSD), ⟨Δ*r* ^2^(Δ*t*)⟩, as a function of lag time, Δ*t*, for polymer networks at different temperatures: (A) *T* = 0.40, (B) *T* = 0.60, (C) *T* = 0.80, and (D) *T* = 1.00. Lengths are scaled by the bead diameter σ. Results are shown for various exchange-attempt intervals, including *t*_Exch_ = 100, 200, 500, 1000, and ∞.

To present a microscopic view of mobility in the networks, we compute the self–part of the van Hove function $G_{s}\left(r,\Delta t=t-t_{0}\right)=\frac{1}{N}\left\langle \sum_{i=1}^{N}\delta \left(r-\left|r_{i}\left(t\right)-r_{i}\left(t_{0}\right)\right|\right)\right\rangle$ , which captures the probability density of particle displacements, at different *t*_Exch_ in Figure 6A,B. We select two time scales, Δ*t* = 10^4^ and Δ*t* = 10^5^, at *T* = 0.8, which cover the spectrum of dynamical modes in these networks, from the suppressed dynamics of the permanently cross-linked network to Rouse-like dynamics of the associative networks. The displacement distributions remain highly sensitive to the exchange-attempt interval scale; faster bond exchange systematically leads to the emergence of an extended tail of *G*_s_ at larger distances, indicating an increased probability of rare long distance hops and thus persistent dynamic heterogeneity^67^ even at high *T*. Already at the shorter lag time (Δ*t* = 10^4^), topology fluctuations facilitate early cage escape events for a subset of highly mobile particles, while at the longer lag time (Δ*t* = 10^5^) the separation becomes more pronounced as bond exchange provides an additional relaxation pathway that broadens *G*_s_(*r*, Δ*t*) but sustains a non-Gaussian, nearly exponential tail, which is a characteristic feature of dynamical heterogeneities in many disordered materials.^68^ In contrast, the permanent network limit (*t*_Exch_ = ∞) exhibits the most rapidly decaying distribution, consistent with strongly constrained motion in a fixed topology, reinforcing that bond exchanges in associative networks primarily modify dynamics rather than the underlying equilibrium structure. Taken together, Figures 5 and 6 show that bond exchange has little effect on short-time local motion but strongly enhances long-time transport and broadens the displacement distribution, indicating that exchange acts as an additional relaxation channel rather than simply accelerating all dynamical regimes uniformly.

**Figure 6:**
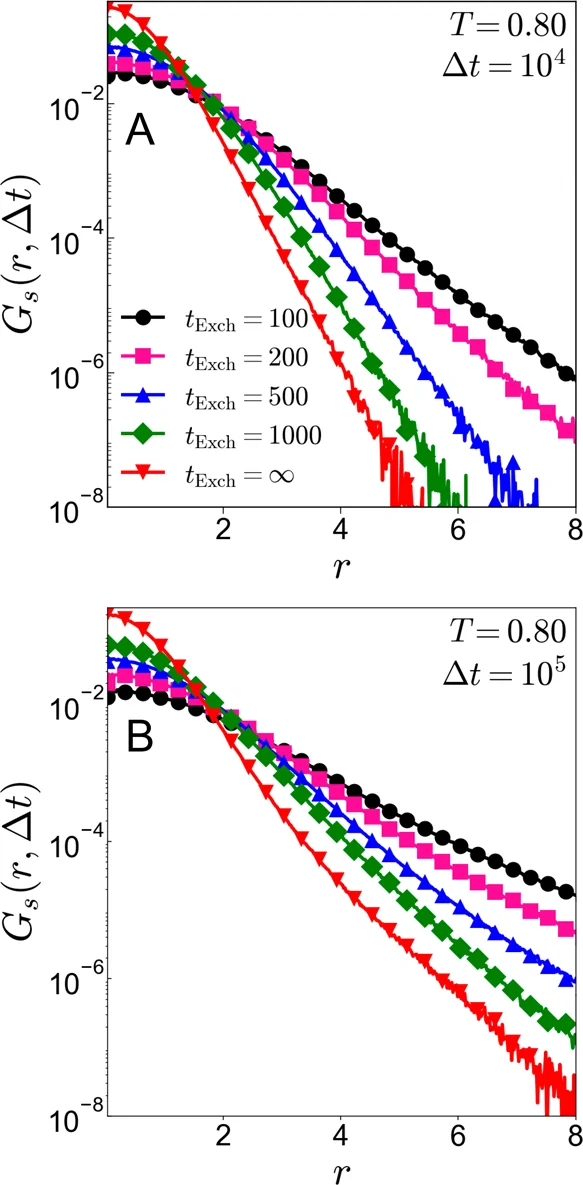
Self-part of the van Hove function, *G*_s_(*r*, Δ*t*), at *T* = 0.80 for different exchange-attempt intervals *t*_Exch_ at (A) Δ*t* = 10^4^ and (B) Δ*t* = 10^5^.

To capture the lifetime of the dynamic bonds, we introduce a binary occupancy variable for each exchangeable bond *b*

3 $$h_{b}\left(t\right)=\{\begin{array}{ll} 1, & \mathrm{if}\;\mathrm{bond}\;b\;\mathrm{is}\;\mathrm{present}\;\mathrm{at}\;\mathrm{time}\;t\\ 0, & \mathrm{otherwise} \end{array}$$

The trajectory therefore defines a time series {*h_b_* (*t*)} for every bond identity *b*. A bond exchange event is detected when a bond that is present in one saved frame is absent in the next, *h*_*b*_(*t*) = 1 and *h*_*b*_(*t* + Δ*t*) = 0, where Δ*t* is the frame spacing. If the same bond identity *b* reappears at a later time *t*′ > *t* (i.e., *h*_*b*_(*t*′) = 1 after at least one frame with *h*_*b*_ = 0), we record this as a rebinding of bond *b*. We characterize the persistence of bond identities using the bond time correlation function

4 $$C\left(t\right)\equiv \frac{{\left\langle h_{b}\left(0\right)h_{b}\left(t\right)\right\rangle }_{b}}{{\left\langle h_{b}\left(0\right)\right\rangle }_{b}}$$

where ⟨·⟩_*b*_ denotes an average over all exchangeable bonds. With this normalization, *C*(0) = 1 and *C*(*t*) shows the probability that a bond present at the reference time remains present at lag time *t*.

As shown in Figure 7, the bond correlation function *C*(*t*) exhibits a clear dependence on both the reduced temperature, *T*, and the exchange-attempt interval, *t*_Exch_. At fixed *t*_Exch_, increasing *T* accelerates the decay of *C*(*t*), whereas decreasing *T* leads to a slower relaxation and a more persistent long-time tail. This behavior indicates that the decay of pairwise bond memory is thermally activated, with higher *T* enhancing segmental mobility and facilitating bond exchange events, while reduced mobility at low *T* kinetically stabilizes the initial bonding configuration. At fixed *T*, decreasing *t*_Exch_ from 1000 to 100 results in a pronounced leftward shift of the relaxation curves, corresponding to a faster decay of *C*(*t*) over the same observation window. Because *t*_Exch_ controls the frequency of exchange attempts, decreasing *t*_Exch_ increases the number of opportunities for successful topology rearrangement and therefore accelerates the loss of bond memory. The realized relaxation time is not set by *t*_Exch_ alone, but also depends on temperature, local geometry, and molecular mobility.

**Figure 7:**
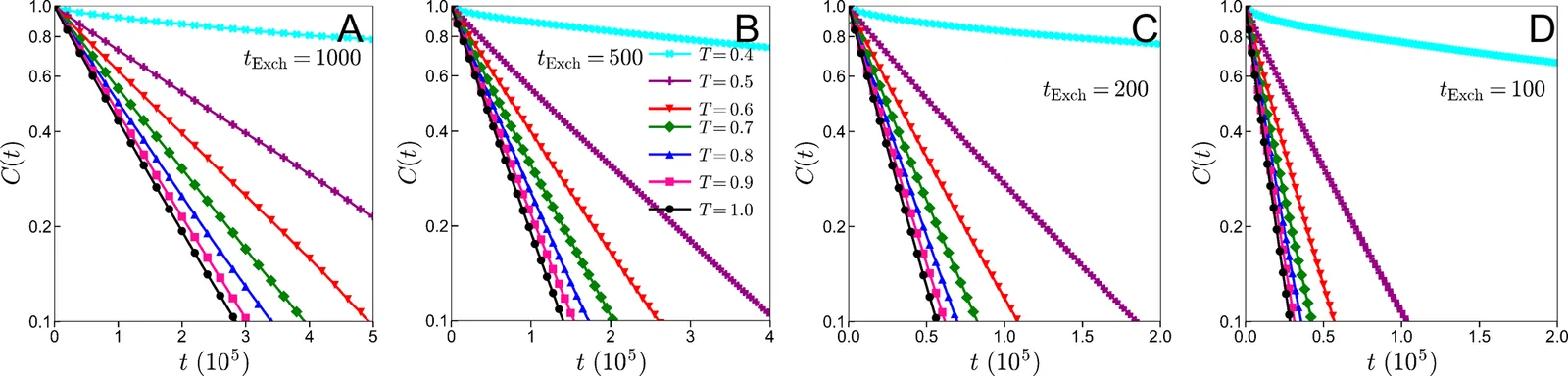
Bond correlation function *C*(*t*) for exchangeable bonds at multiple reduced temperatures *T*, shown for: (A) *t*_Exch_ = 1000, (B) *t*_Exch_ = 500, (C) *t*_Exch_ = 200, and (D) *t*_Exch_ = 100.

Notably, C­(t) becomes less sensitive to t_Exch_ at the lowest (T)*T*, suggesting that thermal constraints on chain mobility can become rate-limiting even when the nominal exchange-attempt interval is reduced. Taken together, these results demonstrate that the relaxation of bond memory in the present system is jointly controlled by thermal activation and kinetic regulation through *t*_Exch_, with faster exchange and higher temperature synergistically promoting more rapid decay of *C*(*t*).

To quantify the decay of bond memory, we define the bond relaxation time, τ_bond_, as the time at which the bond correlation function reaches *C*(τ_bond_) = 1/*e*. Figure 8 shows τ_bond_ as a function of reciprocal reduced temperature for the different exchange-attempt intervals. For every value of *t*_Exch_, ln τ_bond_ increases approximately linearly with 1/*T*, consistent with the increasingly slow decay of *C*(*t*) observed in Figure 7. At fixed temperature, increasing *t*_Exch_ systematically increases τ_bond_, demonstrating that less frequent exchange attempts produce longer-lived bond configurations.

**Figure 8:**
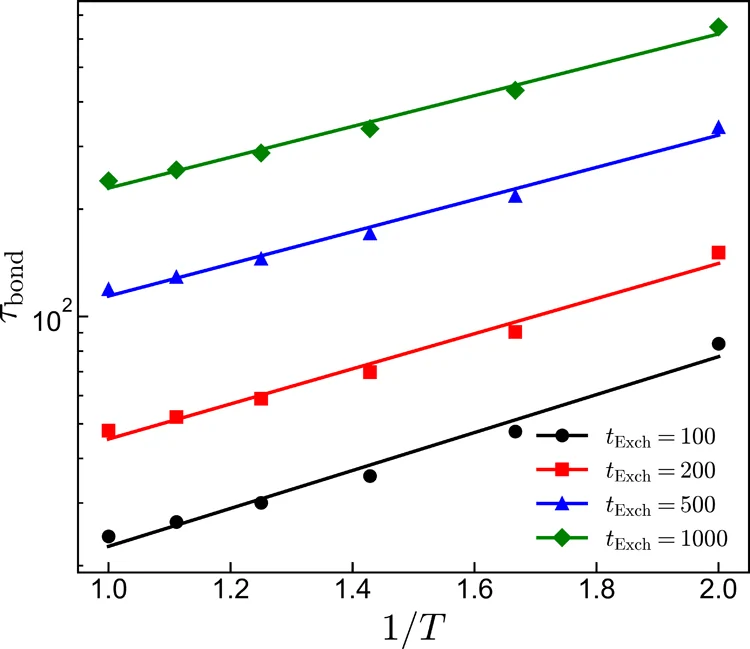
Bond-memory relaxation time, τ_bond_, as a function of reciprocal reduced temperature, 1/*T*, for different exchange-attempt intervals, *t*_Exch_. The relaxation time is defined by *C*(τ_bond_) = 1/*e*. Symbols represent the simulation results, and solid lines are Arrhenius fits of the form ln τ_bond_ = ln τ_0_ + *E*_app_/*T*. The fitted apparent activation energies range from *E*_app_ = 0.908 to 1.225 in reduced units, with *R* ^2^ = 0.979–0.996. τ_0_ follows a linear relationship with *t*_Exch_ according to τ_0_ = 0.086*t*_Exch_.

Over the accessible temperature range, the bond-memory relaxation time is well described by ln τ_bond_ = ln τ_0_ + *E*_app_/*T*, where *E*_app_ is an apparent activation energy in reduced units. The fits yield *E*_app_ = 1.225 ± 0.090, 1.134 ± 0.075, 0.908 ± 0.031, and 0.995 ± 0.052 for *t*_Exch_ = 100, 200, 500, and 1000, respectively. The apparent activation energies vary modestly and nonmonotonically with *t*_Exch_, indicating that the attempt interval primarily controls the overall bond-memory time scale rather than producing a systematic change in its thermal sensitivity. At each temperature, the relaxation times predicted by the Arrhenius fits vary nearly linearly with *t*_Exch_, according to τ_bond_ = *m*(*T*)*t*_Exch_ + *b*(*T*). As the temperature decreases from *T* = 1.0 to *T* = 0.5, the slope *m* increases from 0.230 to 0.599, while the intercept *b* increases from 0.164 to 12.838. Thus, *t*_Exch_ primarily sets the overall scale of bond-memory relaxation, while cooling amplifies the realized bond lifetime through exchange acceptance, local geometry, and molecular mobility.

### 3.3 Creep Compliance

We perform shear creep simulations to quantify how bond exchange kinetics affect network mechanics. The compliance directly reports time dependent deformation and is especially useful for diagnosing terminal flow via the long-time growth of *J*(*t*), enabling systematic comparison across temperature and exchange time. A constant dimensionless shear stress of σ_0_ = 0.1 is imposed, and the resulting shear strain γ­(*t*) is recorded, defining the creep compliance $J\left(t\right)=\frac{\gamma \left(t\right)}{\sigma_{0}}$ .

The creep compliance *J*(*t*) is reported at several reduced temperatures *T* for networks with different exchange-attempt intervals *t*_Exch_ in Figure 9A–D. At *T* = 0.40, which lies below *T*_g_, the shear compliance exhibits only limited growth over the simulated time window, with small fluctuations across all curves that may give the appearance of slight nonmonotonicity, while the overall trend remains increasing. The *J*(*t*) of the permanently cross-linked network shows an equilibrium plateau corresponding to rubbery compliance with increasing temperatures and consistently displays the smallest compliance across all temperatures. On the other hand, the long-time behavior of *J*(*t*) in associative networks at intermediate temperatures *T* = 0.6 and 0.8 shows a combination of a rubbery plateau followed by weak flow. Further increasing the temperature to *T* = 1.0 leads to the appearance of a stronger flow regime in associative networks (Figure 9D), i.e., *J*(*t*) increases more noticeably at long times. The rate of increase of *J*(*t*) is correlated with the bond exchange attempt rate. This trend is consistent with the MSD results discussed in Section 3.2.

**Figure 9:**
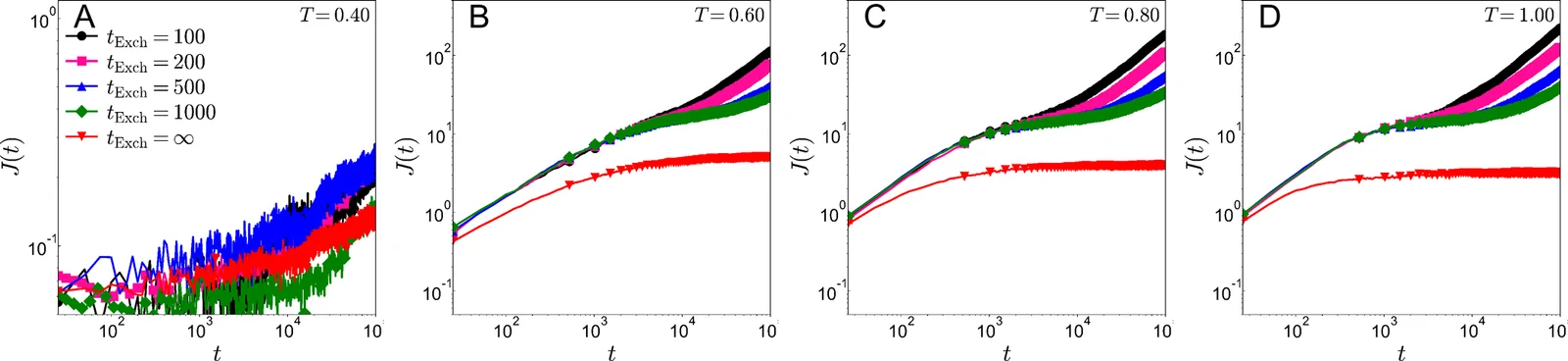
Shear creep compliance, *J*(*t*), at reduced temperatures of (A) *T* = 0.40, (B) *T* = 0.60, (C) *T* = 0.80, and (D) *T* = 1.00 for networks with different exchange-attempt intervals *t*_Exch_. The imposed constant shear stress is σ_0_ = 0.1.

### 3.4 Failure of Time–Temperature Superposition

To assess whether the relaxation dynamics can be described by thermo-rheological simplicity, we performed a TTS analysis using a reference temperature *T*_ref_ = 0.6. In practice, TTS is implemented by rescaling the time axis as *t*/*a*_*T*_, where *a*_*T*_ is a horizontal shift factor. Here, *a*_*T*_ is obtained by manually adjusting the horizontal shift of each curve relative to *T*_ref_.

Figure 10 summarizes the temperature scaling of the MSD and the corresponding TTS analysis after horizontal shifting, while the *a*_*T*_ values are reported in Figure 12A. In the permanently cross-linked network (*t*_Exch_ = ∞), the MSD data in Figure 10A exhibit the expected temperature dependence, with lower *T* slowing down the approach to the long-time plateau. Consistent with thermo-rheological simplicity in this topology frozen limit, these curves can be collapsed onto a single master curve by applying a temperature-dependent horizontal shift, as shown in Figure 10D. The corresponding shift factors *a*_*T*_ reported in Figure 12A are well described by a WLF fit, consistent with a single dominant temperature controlled relaxation clock governed by polymer friction and segmental mobility.^49^

**Figure 10:**
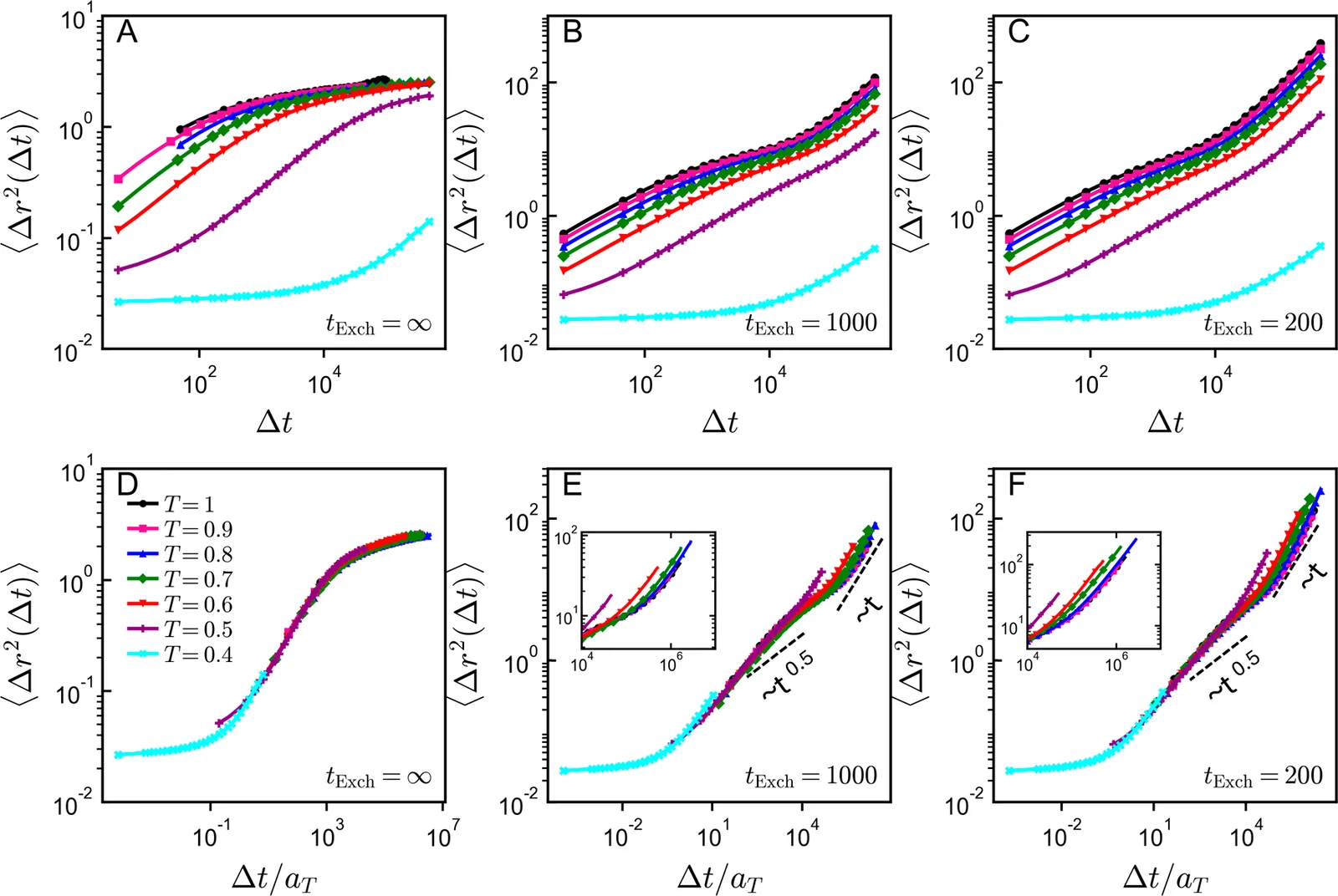
Mean-squared displacement (MSD), ⟨Δ*r* ^2^(Δ*t*)⟩, of cross-linker central beads in vitrimer networks at different reduced temperatures, *T*. (A–C) Raw MSD data for the permanent network, *t*_Exch_ = 1000, and *t*_Exch_ = 200, respectively. (D–F) Corresponding TTS results obtained using horizontal shift factors *a*_*T*_. The insets in E and F show a magnified view of the attempted collapse in the regime where TTS fails.

In contrast, for associative networks with finite exchange-attempt intervals, the MSD does not admit a unique master curve over the full time window. For slow bond exchange rate of *t*_Exch_ = 1000 (Figure 10 B,E), partial overlap can be achieved over intermediate lag times, but the long-time regime cannot be collapsed by a single *a*_*T*_. This breakdown is also seen at a faster bond exchange *t*_Exch_ = 200 in Figure 10 C,F, where exchange mediated transport alters the long-time slope and introduces a crossover whose position shifts with *T* in a way that is not captured by horizontal shifting alone. These results indicate that the MSD contains at least two coupled contributions: polymer-controlled localization and segmental motion, and exchange-enabled topology relaxation. The temperature dependence of these two contributions can now be distinguished directly. The shift factors of the permanently cross-linked network follow a WLF relation, consistent with a polymer-friction-controlled clock, whereas the bond-memory relaxation time in Figure 8 follows an approximately Arrhenius relation. Temperature therefore changes the ratio of the exchange-controlled and polymer-controlled time scales rather than shifting both processes by the same factor.

As this ratio changes, the crossover from localized motion to exchange-enabled long-time transport moves relative to the shorter-time portion of the MSD. A horizontal shift selected to align the polymer-controlled regime therefore cannot simultaneously align the exchange-controlled regime. Consequently, the *a*_*T*_ values obtained from partial overlap of the associative-network curves should be regarded as effective, time-window-dependent shift factors rather than unique material shift factors.

In our previous study of the limiting fast-exchange regime, a broader separation of time scales permitted the identification of distinct WLF- and Arrhenius-dominated regimes and a topology-freezing temperature.^49^ In the present intermediate regime, the two contributions overlap within the accessible time window. The directly measured Arrhenius dependence of τ_bond_, together with the WLF dependence of the permanent-network shift factors, provides a microscopic explanation for the resulting failure of full MSD superposition.

To test whether *J*(*t*) data obey TTS, we construct reduced creep compliance curves using both a horizontal shift factor *a*_*T*_ and a vertical factor *b*_*T*_.^27,28,32^ Specifically, we plot the reduced compliance *J*_p_(*t*; *T*) = *b*_*T*_ *J*(*t*), with the density–temperature vertical shift factor *b*_*T*_, where $b_{T}=\frac{T\rho \left(T\right)}{T_{\mathrm{ref}}\rho \left(T_{\mathrm{ref}}\right)}$ . Figure 11 summarizes the TTS analysis for creep compliance. For the permanently cross-linked network (*t*_Exch_ = ∞), the vertically reduced curves *J*_p_(*t*) differ primarily by a temperature-dependent rescaling of time, and a single pair of shift factors (*a*_*T*_, *b*_*T*_) collapses the data onto a master curve (Figure 11D). The extracted creep shift factors *a*_*T*_ (Figure 12 B) follow a WLF-like temperature dependence, consistent with thermorheological simplicity controlled by a single friction-dominated clock in the permanent network limit.^27,28,69^

**Figure 11:**
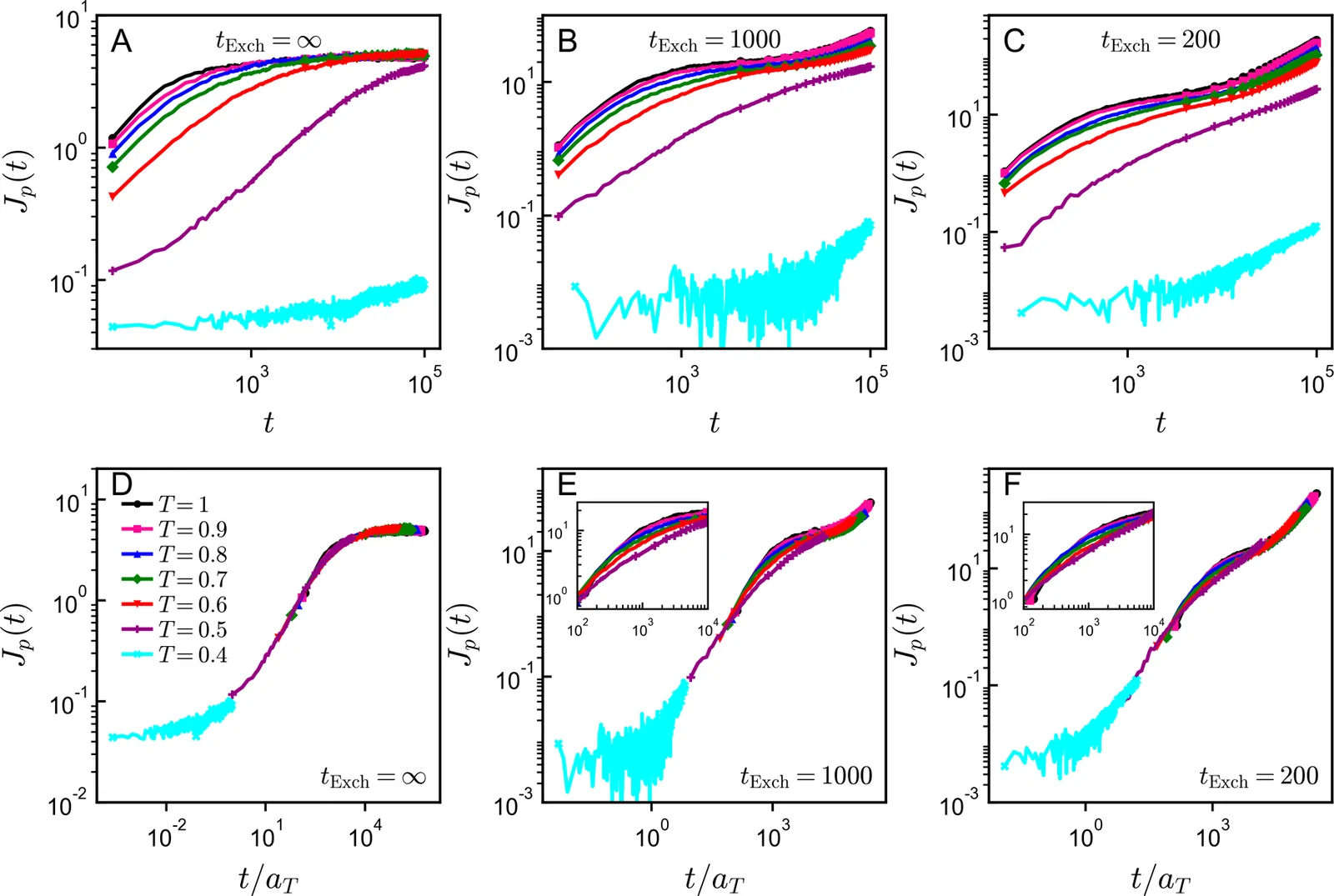
Reduced creep compliance, *J*_*p*_(*t*), of networks at different temperatures, *T*. (A–C) Vertically shifted creep compliance, *J*_*p*_(*t*) = *b*_*T*_ *J*(*t*), as a function of time *t* for three exchange conditions: no exchange (*t*_Exch_ = ∞), slow exchange (*t*_Exch_ = 1000), and faster exchange (*t*_Exch_ = 200). (D–F) Corresponding TTS obtained by horizontal shifting using temperature-dependent shift factors *a*_*T*_ and vertical shifting using *b*_*T*_. The insets in E and F show a magnified view of the attempted collapse in the regime where TTS fails.

**Figure 12:**
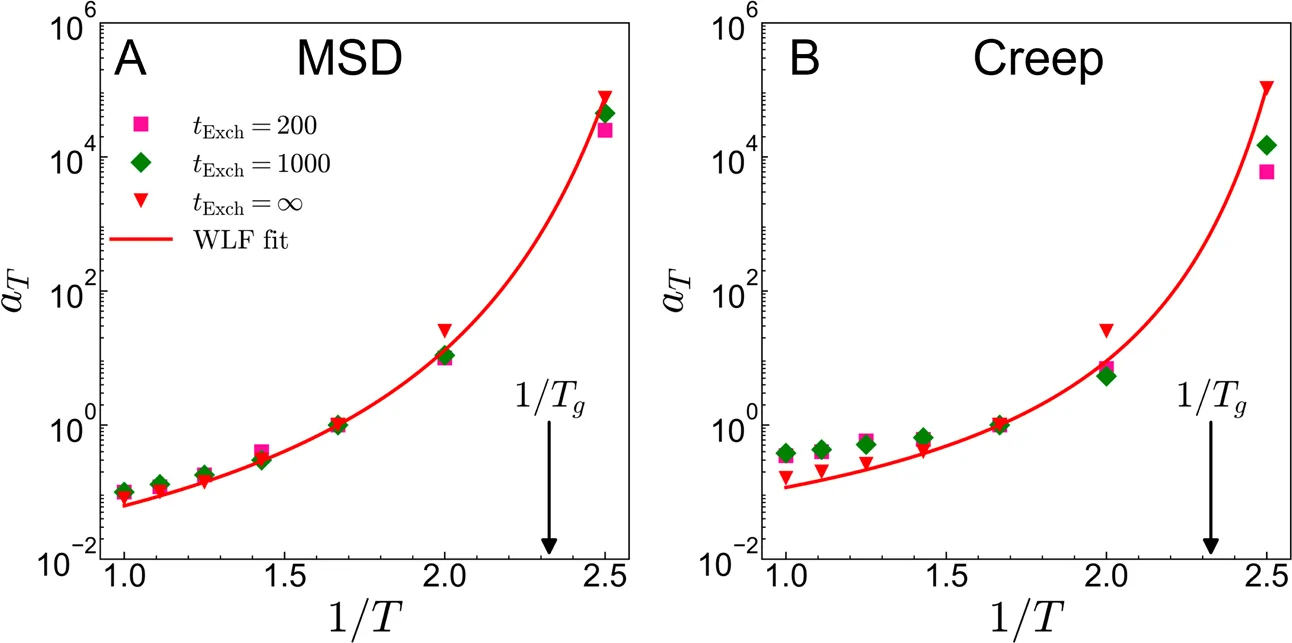
Horizontal shift factors *a*_*T*_ obtained from the TTS analysis of (A) MSD and (B) creep compliance for different *t*_Exch_. The red solid curves are WLF fits to the permanent network (*t*_Exch_ = ∞) only, and the arrow marks 1/*T*_g_. The fitted WLF parameters are *C*_1_ = 2.001 and *C*_2_ = 0.282 for the MSD, and *C*_1_ = 1.560 and *C*_2_ = 0.262 for creep compliance.

In associative networks, creep compliance displays the hallmark features of thermorheological complexity reported for vitrimers: superposition becomes window and mechanism dependent once bond exchange contributes within the accessible spectrum.^25,26,29–31,33,34^ As in the MSD analysis, the apparent shift behavior is similar; a single set of shift factors does not produce a satisfactory collapse across temperatures, consistent with the presence of competing relaxation processes (Figure 11E,F). In particular, the crossover into terminal flow and the long-time growth of *J*(*t*) change with *T*, indicating that the terminal contribution does not share the same temperature scaling as the shorter-time compliance.^28,32^ The direct bond-memory analysis clarifies the microscopic origin of this mismatch. The permanently cross-linked network is governed by a WLF-like polymer-friction clock, whereas exchange-enabled topology relaxation is associated with the approximately Arrhenius τ_bond_ measured in Figure 8. The relative position of these two clocks therefore changes with temperature.

At shorter times, creep primarily probes the elastic and polymer-friction-controlled response of the connected network. At longer times, successful bond exchanges permit topology rearrangement and produce the growth toward terminal flow. Because the WLF and Arrhenius clocks do not shift by the same amount, a horizontal shift that aligns the shorter-time compliance cannot simultaneously align the onset and rate of the long-time exchange-enabled response. This interpretation is consistent with experimental and theoretical descriptions of vitrimer rheology in terms of coupled polymer and exchange clocks.^26,29,30,33^

The *a*_*T*_ values obtained from partial superposition of the associative creep curves should therefore be regarded as effective and window-dependent; they should not themselves be assigned uniquely to a WLF or Arrhenius equation. By contrast, the independently determined shift factors from the permanently cross-linked MSD and creep responses agree reasonably well and follow the WLF eq (Figure 12A,B). The creep results thus provide the macroscopic counterpart of the MSD-based TTS breakdown: the same competition between a WLF-like polymer clock and an Arrhenius bond-memory clock reshapes both microscopic mobility and macroscopic compliance as temperature and exchange-attempt frequency are varied.

## 4 Conclusion

Using coarse–grained simulations of an associative bead–spring network with independently tunable exchange-attempt frequency, we quantified how bond exchange reshapes relaxation while producing only limited changes in the local equilibrium structure. The radial distribution functions remain nearly insensitive to *t*_Exch_, whereas enabling associative exchange produces a modest excess specific volume relative to the permanent network in the rubbery regime. This excess is nearly independent of the finite exchange-attempt interval over the range investigated. Decreasing *t*_Exch_ accelerates the decay of the bond correlation function, shortens the localized or plateau regime of the MSD, and promotes long-time transport without uniformly accelerating short-time local motion.

The direct bond-memory analysis reveals that τ_bond_ follows an approximately Arrhenius temperature dependence over the accessible range, with apparent activation energies *E*_app_ = 0.908–1.225 in reduced units. These activation energies vary modestly with *t*_Exch_. At all temperatures, the bond lifetime increases approximately linearly with the exchange-attempt interval, demonstrating that *t*_Exch_ primarily sets the overall exchange clock, whereas temperature controls the probability of realizing successful topology rearrangements.

These microscopic trends translate directly into macroscopic creep. Faster exchange advances the onset of long-time compliance growth and promotes earlier access to terminal flow-like behavior. More importantly, the combined bond-memory and TTS analyses identify the two relevant temperature-dependent clocks directly. The permanently cross-linked network is governed by a WLF-like polymer-friction clock, while the realized bond-memory relaxation is approximately Arrhenius. Their ratio therefore changes with temperature, shifting the exchange-enabled contribution relative to the elastic, segmental, and plateau portions of the response. This changing separation prevents a single horizontal shift factor from collapsing either the complete MSD or the complete creep response of the associative networks.

The shift factors obtained from partial superposition of associative-network curves should consequently be interpreted as effective, time-window-dependent quantities rather than unique material shift factors. The present simulations provide a microscopic basis for experimental descriptions of vitrimer thermorheological complexity in terms of coexisting polymer and exchange-controlled modes.^26,29,33^ The specific values of the crossover times and apparent activation energies remain model dependent, particularly because the simulated strands are short and unentangled. Nevertheless, the relevant transferable control parameter is the ratio of the realized bond-memory time to the intrinsic polymer-relaxation time, rather than *t*_Exch_ alone. This ratio determines whether exchange-mediated topology relaxation occurs before, overlaps with, or lies beyond the polymer-controlled relaxation window and therefore governs the success or failure of time–temperature superposition.

## Data Availability

The data are available from the corresponding author upon reasonable request.
